# Policymaker, health provider and community perspectives on male involvement during pregnancy in southern Mozambique: a qualitative study

**DOI:** 10.1186/s12884-019-2530-1

**Published:** 2019-10-28

**Authors:** Anna Galle, Helio Cossa, Sally Griffin, Nafissa Osman, Kristien Roelens, Olivier Degomme

**Affiliations:** 1 Department of Public Health and Primary Care, International Centre for Reproductive Health, Ghent University, Corneel Heymanslaan 10, entrance 75, UZP 114, 9000 Ghent, Belgium; 2 Eduardo Mondlane University, Faculty of Medicine, Maputo, Mozambique.Av. Salvador Allende 57, Maputo, Mozambique; 3 International Centre for Reproductive Health - Mozambique, Rua das Flores no 34, Impasse 1085/87, Maputo, Mozambique

**Keywords:** Male involvement, Maternal health, Male involvement policies, Mozambique

## Abstract

**Background:**

Increasing male involvement during pregnancy is considered an important, but often overlooked intervention for improving maternal health in sub-Saharan Africa. Intervention studies aimed at improving maternal health mostly target mothers hereby ignoring the crucial role their partners play in their ability to access antenatal care (ANC) and to prevent and treat infectious diseases like HIV and malaria. Very little is known about the current level of male involvement and barriers at different levels. This study explores the attitudes and beliefs of health policymakers, health care providers and local communities regarding men’s involvement in maternal health in southern Mozambique.

**Methods:**

Ten key informant interviews with stakeholders were carried out to assess their attitudes and perspectives regarding male involvement in programmes addressing maternal health, followed by 11 days of semi structured observations in health care centers. Subsequently 16 focus group discussions were conducted in the community and at provider level, followed by three in depth couple interviews. Analysis was done by applying a socio-ecological systems theory in thematic analysis.

**Results:**

Results show a lack of strategy and coherence at policy level to stimulate male involvement in maternal health programmes. Invitation cards for men are used as an isolated intervention in health facilities but these have not lead to the expected success. Providers have a rather passive attitude towards male involvement initiatives. In the community however, male attendance at ANC is considered important and men are willing to take a more participating role. Main barriers are the association of male attendance at ANC with being HIV infected and strong social norms and gender roles. On the one hand men are seen as caretakers of the family by providing money and making the decisions. On the other hand, men supporting their wife by showing interest in their health or sharing household tasks are seen as weak or as a manifestation of HIV seropositivity.

**Conclusion:**

A clear strategy at policy level and a multi-level approach is needed. Gender-equitable relationships between men and women should be encouraged in all maternal health interventions and providers should be trained to involve men in ANC.

## Background

Antenatal care (ANC) plays a critical role in the health of pregnant women worldwide and is considered as a key entry point to receive preventive health care including nutritional support, prevention and treatment of several diseases (malaria, tuberculosis, neonatal tetanus, syphilis and HIV), as well as identification and management of potential complications during pregnancy [1, 2]. Moreover, during ANC visits women can receive counselling about family planning methods and postpartum care for themselves and their newborn [3]. In Mozambique, 93.3% of women go for at least one ANC visit during pregnancy, but only 54.6% receive the recommended four ANC consultations [4]. ANC services are provided free of charge but clinical, social, economic, and cultural barriers limit access to high quality ANC. These barriers include transportation problems, lack of social and financial support from family members and distrust in the health care system [5, 6]. Women are socially expected to ask permission from their male partners before making decisions about their own health care utilization [6]. Besides the husband, other key actors in the referral of pregnant women to the health services in southern Mozambique include matrons (influential older women), community health workers (CHWs), and neighbors [6]. Lack of spousal permission and fear of going to the clinic alone represented half of the reported barriers to ANC uptake in a national survey [5, 7].

Most interventions to improve maternal health and ANC uptake target mothers even though partners play a crucial role in women’s ability to seek and obtain better antenatal care, to prevent and treat HIV infection that contributes to maternal mortality, and to reduce the incidence of obstetric complications [8]. Research in sub-Saharan Africa has demonstrated that fathers’ involvement during pregnancy is associated with positive outcomes for the mother and baby, which include more antenatal care visits, participation in strategies to prevent vertical Human Immunodeficiency Virus (HIV) transmission, increased institutional delivery and better birth preparedness in case of pregnancy complications [9–15]. The signatories of the International Conference on Population and Development Programme of Action plan emphasized in 1994 that it is important for men to take more responsibility for their sexual and reproductive behavior and family life, and proposed that countries outline the responsibilities, plans and strategies for involving men in sexual and reproductive health [16]. In 2015 The World Health Organization (WHO) also recommended to include men in MCH (maternal and child health) programs in their recommendations on health promotion interventions for maternal and newborn health [17]. WHO states that male involvement interventions should be implemented in a way that respects, promotes and facilitates women’s choices and their autonomy in decision-making and supports women in taking care of themselves and their newborns. Interventions to involve men in maternal health have been delivered through diverse mechanisms including community outreach and education, mass media social mobilization campaigns, the use of invitation cards for men to attend ANC, education for men only or for men and women together, home visits, and facility-based counselling for couples, groups or men only [15, 17].

Existing studies define the concept of ‘male involvement’ differently, indicating it is a subjective and multifaceted term [8, 11, 18, 19]. Most studies define male involvement as one or a combination of the following elements: active participation in maternal health services and care, financial support given for pregnancy-related and childbirth-related expenses and shared decision-making powers regarding maternal health issues [8]. While several studies have explored the role of partners in ANC uptake and engagement, this has not yet been explored in southern Mozambique. Research in sub-Saharan Africa showed that men perceived an unfriendly clinical environment and the negative attitude of providers as major barriers towards their engagement in maternity care [20]. Audet et al. (2015) examined male involvement in ANC in central Mozambique and two main barriers to increased male involvement in maternal health emerged: (a) gender inequality in decision making and (b) community beliefs that uptake of ANC services, particularly if supported by a male partner, reflects a woman’s HIV-positive status. However, in general, southern Mozambique is significantly different socio-economically and culturally compared to the north and centre of the country, including in terms of gender dynamics and, consequently, facilitators and barriers to male involvement are likely to be different. Northern and parts of central Mozambique have a matrilineal marital, kinship and inheritance system while Southern Mozambique has a patrilineal system [21]. In a patrilineal system the woman moves to live with the husband’s family after marriage, that pays a bride price to the woman’s family in exchange. In this system women often have less power than in a matrilineal system because the man has “paid” for the woman and thus has power over her and their children [21].

The overall goal of this research was to explore the attitudes, practices and beliefs of health policymakers, health care providers and local communities regarding the benefits, challenges, risks and approaches to increase men’s involvement during pregnancy in southern Mozambique.

## Methods

### Setting

The study was carried out by Ghent University in collaboration with the International Centre for Reproductive Health – Mozambique (ICRH-M) and Universidade Eduardo Mondlane (UEM) between March and October 2017. ICRH-M is a Mozambican Non-Governmental Organization (NGO) and research institution. UEM is the main public university in Mozambique.

The study was conducted in Marracuene and Manhica districts in Maputo Province. This region has around 334,000 inhabitants and 21 rural health centers. This study site was involved in previous ICRH-M studies, and therefore health providers and health managers working in this area have a constructive relationship with the principal investigator and ICRH-M researchers.

### Research team

The principal investigator (AG) is a Belgian doctoral student with research experience in Mozambique. She was assisted during all focus group discussions (FGDs) and couple interviews by a research assistant (HC) and two fieldworkers. The research assistant was a final year medical student doing an internship at the reproductive health research unit of the Universidade Eduardo Mondlane. Key informant interviews (KIIs) and observations were conducted by the researcher alone prior to the FGDs.

### Participants & study procedures

Data collection took place between March and October 2017. Firstly 10 key informant interviews were carried out over a 2 month period, followed by 11 days of semi-structured observations as preparation before the FGDs. Afterwards 16 FGDs were conducted with providers and community members to explore different aspects of male involvement, which were explored further in three in depth couple interviews. FGDs were spread over a period of 5 month period to allow for minimal interim analysis, followed by the in depth interviews.

The key informant interviews were conducted with maternal health policymakers, researchers and NGO staff. Men and women specialized in maternal health policies, program implementation and research in Mozambique were eligible for participating in the interviews, which aimed to frame the topic within the political and structural context of Mozambique. Experts working in private clinics or commercial organizations were excluded. Participants were identified using a ‘snowballing’ approach. The first round of contacts was identified by personal contacts of the authors (AG, OD, SG, NO and KR) and reviewing attendee lists of national maternal and child health conferences. In addition interviewed contacts were asked to nominate other appropriate key informants. Key informant interviews all took place face-to-face in a private room. All interviews were recorded and transcribed, except for one interview where only notes were taken (no recording was allowed by the participant).

Subsequently, AG conducted sit-in observations at antenatal clinics and attended health promotion sessions at the different study sites to explore the workload of providers, number of men attending ANCs, power dynamics within the consultation and quality of care provided. Seven different study sites were purposively selected to include health centers with different characteristics (high versus low workload, urban versus rural). Both a checklist (see Additional file 1) and written narratives were used to collect the data of the observations. In total 159 antenatal consultations were observed.

Afterwards, FGDs were conducted with providers and community members. For the FGDs with providers, the heads of the health centers were contacted to discuss a date and time for conducting the FGDs. All health care centers in Manhica and Marracuene were listed and the study sites were purposively selected to include health centers with different characteristics and good road access. FGDs were planned during lunch break or after working hours. Men and women were mixed in the focus group discussions with providers, as we believed in this group the gender dynamics are of interest and would not affect the openness of the participants. For the FGDs in the community, community leaders were contacted in advance with an invitation letter. Communities with different characteristics were selected (distance to a health facility, rural versus urban, seasonal wave of work or year-round employment, …) for the study. All respondents for the FGDs in the community were purposively selected to represent certain segments of the population—namely, pregnant women or pregnant < 2 years ago, male partners, community leaders, health activists, CHWs, and traditional birth attendants. FGDs in the community were divided into male and female groups, since we believed this composition may make participants more likely to discuss topics openly together than if groups were mixed. FGDs were conducted in a private place, inside the community office or under a tree away from other activities, and the date and time were decided by the community leaders. For both provider and community FGDs, the number of participants per group ranged from 5 to 8. FGDs in the community were conducted in the local language (Changana), while provider FGDs were conducted in Portuguese. All FGDs were facilitated by the researcher, assisted by two local fieldworkers. The researcher is fluent in Portuguese and the fieldworkers were fluent in both Portuguese and the local language. FGDs in the community with men were assisted by a male fieldworker and FGDs in the community with women by a female fieldworker. FGDs were spread over a period of 5 months to allow for minimal interim analysis. Data was collected until data saturation. The interview guides for the key informant interviews and FGDs can be found as additional files (see Additional file 2).

Subsequently, themes that emerged during the community FGDs were discussed with three couples, the couples were purposively selected in order to include couples at different stages of their reproductive life. As FGDs within the community were conducted with men and women separately, we wanted to conduct these interviews with men and women together to generate new insights regarding the dynamics within couples. In depth couple interviews were conducted in the local language (Changana).

### Data analysis

**Fig. 1:**
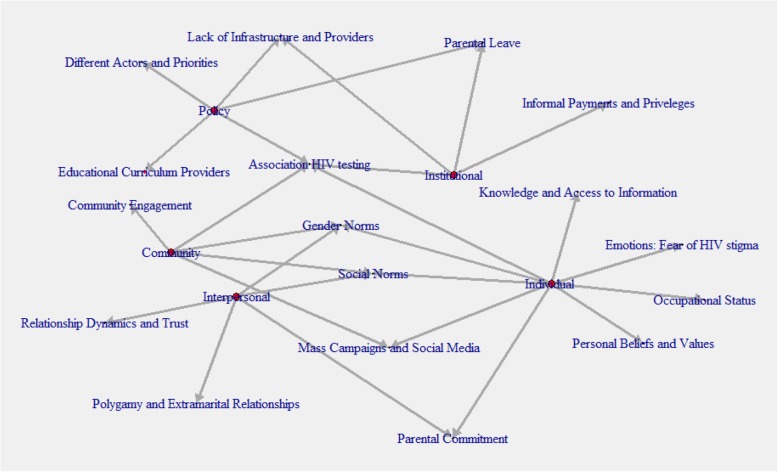
Socio-ecological framework with emerging themes in RQDA-plot

All interviews and focus group discussions were transcribed verbatim in Portuguese, except for the FGDs conducted in the local language, which were translated into Portuguese during transcription. Transcription from the local language into Portuguese was conducted by the research assistant (mother tongue Changana) and an extra interpreter as a double check. Thematic analysis was used as data analysis method and the framework approach was used as a tool, including 7 stages of analysis: transcription, familiarization, coding, developing a working analytical framework, applying the analytical framework, charting data into the framework matrix, interpreting the data [22]. R Qualitative Data Analysis (RQDA) software was used for coding. All data was coded by both AG and HC, afterwards all codes were discussed, and they agreed on a set of codes and categories (first four steps of the framework analysis). After reviewing a number of theories on access to healthcare and health promotion programming [23–27] the socio-ecological framework was identified as the most appropriate model to guide the analysis. Four units of analysis were identified: individual, interpersonal, community and health system related factors (see Fig. 1). The last three steps of the framework approach were supervised by OD. Field notes from observations were also analyzed and added as analytical memos that facilitated interpretation of certain phenomena that emerged during data analysis. All analysis was carried out in Portuguese and some quotations were translated into English by the researcher in the present article. Only age ranges were reported along with quotations to guarantee anonymity. Translations of quotations were double checked by a bilingual colleague (mother tongue Portuguese).

### Ethical considerations

For the observations all health directors and health providers of the participating health centers were asked for permission after explaining the aim and procedures of the study. The women and their partners (if present) were asked orally if they consented to be observed during consultation before entering the consultation room. The provider explained to them the observer was a Belgian midwife, conducting a study about the Mozambican health system.

For the FGDs with providers the district officers were contacted for authorization and organizing the FGDs. FGDs in the community were organized by the community leaders. Information about the objective of the study and procedures was provided to all respondents (KIIs, FGDs and in-depth interviews) orally and in writing. Participants were asked if they consented to interviews being recorded using a tape recorder. Confidentiality, anonymity and ground rules were discussed before starting an interview or FGD. Participation in the study was voluntary and all participants of the key informant interviews, FGDs and in-depth interviews gave their written consent. Participants not capable of signing could provide their fingerprint. No incentive was provided, other than refreshments during FGDs. Ethical approval for the study was obtained by the medical ethical commission of Ghent University (EC/2018/1319), the National Health Bioethics Committee of Mozambique and the Health Bioethics Committee of UEM and HCM (Maputo Central Hospital) (CIBS UEM&HCM/0008–17).

## Results

### Semi-structured observations

The partner was present in 4.4% (*n* = 7) of the observed consultations. 23.3% (*n* = 37) consultations were first ANC consultations, during which approximately one third of women (36.1%; *n* = 11) received an invitation card for the partner. Of the women coming for a follow up ANC visit, 9.4% received an invitation card.

All women that came for their first ANC (*n* = 37) were tested for HIV, six of these women were HIV positive, and of these, five women received the invitation card for their partner.

### Key informant interviews

Ten key informant interviews were conducted with maternal health experts from different fields (see Table 1). Age of the respondents varied between 42 and 54 years old.

**Table 1:** Participants’ characteristics

	Male	Female
Key informant interviews (n = 10)		
Key informants background		
∙ Government (MoH)	0	3
∙ NGO	2	2
∙ Academic background	1	2
Community FGDs (n = 10, total 63 participants)		
Position in community		
∙ Activist/CHW	1	6
∙ Child <2 years old	6	15
∙ Pregnancy	4	7
∙ Community Leader	11	6
∙ Traditional Midwife	0	5
∙ Traditional Healer	1	1
Level of education		
∙ None	1	8
∙ Primary	20	27
∙ Secondary	2	2
∙ Higher education	0	1
Marital status		
∙ Single	3	14
∙ In relationship	20	21
∙ Widow	0	4
Providers FGDS (n = 6, total 36 participants)		
Function in health center		
∙ MCH nurse	0	13
∙ General nurse	2	1
∙ Technical Pharmacist	0	3
∙ Technical Agent	2	9
∙ General Doctor	0	1
∙ No qualification	1	1
∙ Social worker	1	5
Marital status		
∙ Single	4	18
∙ In relationship	1	15
Couple interviews (n = 3, total 6 participants)		
Reproductive life stage		
∙ Pregnant couple expecting first child	1	1
∙ Young couple with 6 children	1	1
∙ Senior couple with grandchildren	1	1

### FGDs

In total 38 providers participated in 6 focus group discussions organized at 6 different health facilities (Table 1).

At the community, 10 FGDs took place in four different communities, with a total of 63 participants. Average age of the community participants was 38 years old and the average number of children 4. 64% of the respondents was female (Table 1).

### In-depth interviews with couples

Three in-depth couple interviews were carried out. The first couple was a young unmarried couple expecting their first child. The second couple was a community leader with his second wife and five young children and the last couple was a senior religious leader with his wife.

#### Individual-level barriers/facilitators

### Personal beliefs and values

At the individual level it was clear that men are interested and feel responsible for their partner and unborn baby but that they express their involvement in ways other than going with their wife to ANC. Some men did see it as their task to accompany their wife inside the consultation, but these cases were rather exceptional. In general the younger generation was especially interested in what happens during the ANC consultation.

*“I went with my wife to the antenatal consultations but I had to wait outside, although I would have liked to know what happens inside. I also want to accompany her for delivery but it’s not allowed.” Expectant father, age group 30–40, couple interview.*

Most men assist with logistics, money and accompaniment to the health facility gate. Health care issues and especially pregnancy and childbirth are considered a female domain by the majority of men. When someone in the family (such as wife, baby or other relative) is seriously ill the man will assist by accompanying them to the hospital and will take part in decisions, but for regular visits it is considered a waste of their time. The few men that considered male attendance at ANC to be important explained that, for them, this support is a part of taking care of your family and loving your wife.

*“I think it’s important men are involved and go with the wife to ANC because … it’s an act of showing love.” Male religious leader, age group 50–60, community FGD.*

### Knowledge and access to information

Most men in the community have limited knowledge about ANC or maternal health. In the male FGDs much more framing and explanation was needed about the aim of the discussion, because the participants often did not really know what an antenatal care consultation is, considering all consultations to be the same. This lack of knowledge may mean that men also have limited knowledge about pregnancy and may be resistant to any health behavior or recommendation during pregnancy (such as male involvement, or the importance of good nutrition or a bed net).

### Emotions: fear of HIV stigma

Male FGD participants stated they have to accept an HIV test when they go to the clinic, and that most men are scared of the result and associated stigma. HIV is often seen as a women’s disease, so a lot of men believe that women should deal with it. Female respondents explained women are often blamed for an HIV infection. When the woman gives her partner the invitation card from the health center, this can create conflict because it is associated with HIV positive couples and bad news. Women are reluctant to invite their partner because they are afraid of being abandoned if they test positive during the consultation. A lot of women do not reveal their status and take anti-retroviral medicine (ARVs) without their partner being aware because of the emotions and conflict this might create between the couple.

*“The good thing is, when a pregnant woman tests positive, she might not tell her husband but at least she will take the ARVs. She will just do it in secret. That’s why they never want to bring their husband, they are afraid he will discover and abandon them once they know she is HIV positive.” Female Provider, age group 20–30, Provider FGD.*

Providers also explained that a lot of men do not want to be involved in the treatment of “women diseases” such as those associated with HIV, pregnancy and childbirth. However, they also mentioned that men can become more engaged in these issues when the importance of treatment or care for the unborn child is emphasized.

*“Men are more interested in the health of the baby than the health of the woman. The wife they can change anytime but the baby will be theirs forever.” Female Activist, age group 30–40, Community FGD.*

### Occupational status

A lack of time was one of the reasons most cited by men for not attending ANC with their wives, compounded by the risk that a man may lose a day of income when he chooses to accompany his wife. However, when this theme was discussed in the in-depth interviews men revealed that this may be more of an excuse than a real barrier. They explain that they see it as a matter of priorities, since most men have spare time to spend on activities outside work, and since most employers accept a temporary absence from work for this occasion. Their perception is that the main barrier is that men consider their attendance at ANC as “a waste of time” rather than the fact that they cannot leave work.

*“To be honest, it’s not a matter of time. You will see men outside drinking with their friends. Those are the same men that say that they don’t have time.” Father, age group 30–40, couple interview.*

#### Interpersonal barriers/facilitators

### Relationship dynamics and trust

Both men and women in the community explain that expecting a child can change the relationship dynamics within a couple. During pregnancy negative feelings of uncertainty, neglect and distrust are more common. Intimate partner violence as a consequence of the stress related to pregnancy was mentioned by both men and women. For some men the stress around pregnancy is a trigger to abandon their wife, initiate extramarital relationships or start beating their wife. Domestic violence when there are frustrations is still common, but according to older community members it is less frequent in the younger generation. There was a general consensus among community participants that intimate partner violence is unacceptable and that taking care of the wife should be the norm.

These changing dynamics and distrust in each other during pregnancy might explain why many men and women mention that lack of trust is one of the main motivators for men to be involved during pregnancy. Men think their pregnant wife might not actually go to ANC or might lie about recommendations of the provider. Additionally, providers often say that men should accompany their partners in order to receive the information “first hand”. Consequently, a lot of men believe there is no need to accompany their pregnant partner to ANC if they can trust her, in which case they assume she will give them any important information after the consultation.

“*If you don’t trust your wife it’s better to go with her. If you trust her, you can let her go alone”. Male community leader, age group 50–60, Community FGD.*

Therefore male involvement motivated by distrust is not always considered as positive for women.

“*For those women who have a good, open and honest relationship with their husband it’s fine to bring their husband. For those who are married to the … euhm the typical macho man, it’s difficult, they are not open, there is no trust. They are scared to invite their husband, to bring their husband … They are scared to be accused of HIV infection.” Female provider, age group 30–40, Provider FGD.*

Furthermore, when couples go together to ANC, very often the man takes the lead in terms of the discussion and decisions. A provider explained:

*“Women can take the initiative, but the man has the last word.” Female provider, age group 30–40, Provider FGD.*

Many providers stress the importance of male involvement and male attendance at ANC during pregnancy for the relationship with the mother (psychosocial support) and the benefits for the unborn child. They explain that participating male partners have a better relationship with their child in the future compared to partners that are absent during pregnancy. Also women explained that they want their husbands to be present because it reduces their stress. Men do want to support their partners but in general that think it is more important to support her with practical arrangements such as good food, money and housing than with psychosocial support.

In relation to money, the providers and community members explain that women receive a small part of the men’s salary for household tasks and food. Most women are allowed to go to ANC by their partner and can use some of the household money for transport. For health related decisions that represent a considerable cost (for example transfer to a referral hospital) she needs authorization of her husband, or in his absence someone from his family will take the decision.

### Polygamy & extramarital relationships

In situations of polygamy the relationship can become complex. When a man has more than one wife it becomes hard to be involved in each pregnancy, for example by going to ANC with each spouse. Both providers and women explain that polygamy often causes problems because the man cannot take care of the different wives at the same time.

*“Mostly when the man is not very involved or is absent it’s because he has another wife somewhere. If he takes a second wife he should take care of them equally. But this is not easy. If one receives meat, the other one will complain if she is not receiving the same.” Male community leader, age group 50–60, Community FGD.*

### Parental commitment

An important factor that will influence a man’s decision to be involved is accepting responsibility for the pregnancy. Especially when it is the first pregnancy he might try to “escape” from his responsibility and claim the unborn baby is not his child. Whereas when the pregnancy is planned men are usually proud of their unborn child and will more willingly engage, this is less common when the pregnancy is unplanned, when there is a high risk the man will not accept the pregnancy. Community, members describe this as something that happens more often than before, since more relationships are informal (not officially married/living together) which makes it easier for men to disown the pregnancy.

*“When we were young, sex before marriage didn’t exist. The problem is that now youngsters start with sex even before knowing each other. When the girl gets pregnant the guy just runs away.” Male community leader, age group 50–60, Community FGD.*

Before, parents were more involved and the parents of the pregnant girl would negotiate with the man’s parents to accept the pregnancy and to marry the girl. Nowadays the leadership role of the parents and elders is less strong in the communities. It is common for women to be abandoned by their boyfriend once the pregnancy is discovered, although sometimes a man who has left his partner during pregnancy comes back when the child is born. This is mostly a couple of months after delivery, when the most difficult and risky period has passed and when the father can interact and play with the child. Providers explained that male involvement is more common in pediatric consultations because of these reasons.

*“A husband will seldom show interest in his wife when the baby is still in the “cervix”, when the baby is out of the cervix, yes he will come.” Female community health worker, age group 30–40, Community FGD.*

#### Community barriers/facilitators

### Gender norms

Strong gender norms persist in the community. Men, women and providers expressed the opinion that a man should not waste his time queuing and that men get priority at the health facility to receive care. They justify this rule by explaining that men often have work responsibilities. The man takes the lead in daily life (e.g. opening the door for visitors, negotiating prices and constructing a house for the family) and this dominant role is also evident when the man goes with his wife to ANC. If a man is present in the ANC consultation room, the consultation will be directed towards the man, who having received information there will then take care of his wife by giving her the medication or supervising her behavior. This is not perceived as incorrect or discriminatory by either women or providers.

Providers, men and the majority of women consider the priority rule for men as a positive initiative. Only one male community leader and one traditional midwife were critical of this priority rule and the associated gender inequality between men and women.

*“In the bible it’s written that we are all equal, man and woman. But a lot of men don’t consider women as equal. A woman can have five children and will take care of all of them. You will hardly find a man who will take his responsibility and take care of his children. Even if he has just one child, he will leave the kid with a sister or his mother and leave. He might only return the next day, after going out drinking. A woman on the other hand will combine everything. She will study, work and take care of all her children.” Traditional midwife, age group 40–50, Community FGD.*

### Social norms

Supportive men are considered as “good” men in the community but this support should not be expressed by doing “female tasks”. Most men are afraid of the reaction in the community when they accompany their wives to ANC or help in domestic work. They explained that embarrassment is one of the most pertinent barriers to their engagement in maternal health issues. A supportive husband works outside of the home and takes care of his family, but does not necessarily go with his wife to ANC. The majority of community respondents did not like this strong distinction in male and female roles but at the same time felt pressure from the community to respond to these traditional masculinities. Men are afraid that people will laugh at them or think their wife “bewitched” them when they accompany her to ANC.

*“If you see a man going with his wife most people think he has HIV or has another serious disease. Or he is not good in his mind.” Young father, age group 20–30, Community FGD.*

Some older men also believe it is not appropriate to enter inside the ANC room.

*“Yes we will bring her to make sure she goes to the consultation. But going inside, no, that’s not for us.” Male religious leader, age group 50–60, Community FGD.*

On the other hand, providers were of the opinion that everyone in the community knows that men’s involvement in pregnancy is important and that these social norms are no longer a major barrier. They make the comparison with homebirths, in that nowadays almost everyone accepts that it is better to deliver in the facility. They believe that social norms are constantly changing, influenced by what is advised at the health facility and during community health promotion talks.

### Community engagement

Many providers stress that more effort and initiative has to come from the community. They suggest that by having some “champions” or “good examples”, others can adopt these practices. Both the community and providers recommend investing more in health talks in the community and organizing these for a mixed audience of both men and women.

*“At the health care center we do enough. We give health talks, give invitations for men and they don’t have to wait in the queue. Now the effort has to come from the communities.” Female Provider, age group 20–30, Provider FGD.*

Another practical recommendation that was made is to address male attendance at ANC during local community meetings because these are mostly attended and organized by men (community leaders).

*“We have monthly community meetings. You only see men there. That’s where they should talk about male involvement.” Male community leader, age group 40–50, Community FGD*.

Providers also stress that the focus should be on male involvement in all health issues (including general consultations, pediatric care, and antiretroviral therapy) and not only on one issue because this can lead to misunderstanding (such as associating male attendance at ANC with HIV status).

### Mass campaigns & social media

A lot of men reported being informed about the importance of health care issues through radio, television and community mobilization programs. However, these initiatives often focus on spreading general health information and do not focus on the active role fathers can play during pregnancy and childbirth.

Providers reported seeing increased interest by a small number of men and believe this is because of public service announcements on television and wider access to the internet, as opposed to the efforts at the health center. Young men see role models involved in family life on television and by WhatsApp internet/messages, which stimulates this behavior change.

*“But there is already more access to information and that helps. Men take better care of their wife than before. For example, on television, before and after the news you have publicity where you see men involved in the family.* “*Male provider, age group 30–40, Provider FGD.*

#### Institutional

### Lack of adequate infrastructure & competent providers

At all levels there is consensus that the health system is not set up to receive men. Important health checks such as blood pressure screening and weight control are often skipped because they are time consuming and appropriate material is unavailable. There is a lack of privacy during ANC because of the poor infrastructure and high work pressure, which is felt to be less of an issue with women-only consultations. Frequently two consultation are done simultaneously in the same room, and even for intimate exams or HIV testing privacy cannot be guaranteed. If more men are present at ANC this will become even more complicated.

Community members explained that it depends on the provider whether or not a man can enter the ANC room. Providers claim they always invite the man inside but during the observations it was evident that some providers indeed prefer to see the woman alone in order to gain time. Only after the consultation they will invite the husband inside for an HIV test.

Both men and women in the community have a high trust in the competence of the provider and their advice or expertise will almost never be criticized or questioned. Providers explained that the majority of men think that if they bring their wife to the hospital they are “saved” and that they have done their job.

*“Men don’t prepare anything before the delivery because of myths. It brings bad luck to prepare something before the baby is born. But men also think if they drop the wife at the hospital for delivery everything is fixed. Health care providers are almost “God”.” Female provider, age group 20–30, Provider FGD.*

For delivery both providers and community participants stated that the man can bring his wife to the health center but will be sent away to wait at home for news. None of the respondents mentioned that men had ever been allowed to accompany their wife for the delivery. Several men and women in the community would be in favor of permitting the husband as a companion during labor and/or delivery. Currently only female companions are allowed.

### Informal payments and privileges

Both men and women in the community explain that payment of informal fees is very common and this was confirmed during the observations. Community leaders explained that informal fees are almost standard for care during delivery, and are hidden in the woman’s sarong (*capulana*). They explained that extra payments are often made for receiving medication, better treatment and allowing a companion during delivery. During ANC, informal fees are less common but can also happen, mostly in order to obtain better/faster treatment or medication. Women need to ask for money from their husbands to pay for these privileges and as this practice is widely known, men will support their wife with extra money if they can afford it.

*“You know, here you have to pay for everything. That is the biggest problem in Mozambique. For delivery you will have to pay if you want your wife to be treated well and have a birth companion. You pay or she might deliver alone.” Male community leader, age group 40–50, Community FGD.*

### Association with HIV testing

Although it is not official policy, all providers explain that everyone is obliged to get tested for HIV at the health center. Male accompaniment during ANC is highly associated with HIV testing. In the community it is common knowledge that if you go to ANC you will have to accept an HIV test. This idea stems from previous Prevention of Mother to Child Transmission of HIV (PMTCT) campaigns where men were encouraged to go to the health center for HIV testing.

*“Here everyone gets tested, it’ obligatory. Officially maybe it’s not obligatory but in this health center nobody leaves the antenatal consultation without testing.” Female provider, age group 30–40, Provider FGD.*

#### Policy level

### Different actors & priorities

At policy level all experts explained that there is no clear written strategy at national level regarding male involvement in maternal health. Currently two interventions are being implemented in ANC: the distribution of invitation cards for men and the priority rule for couples. Interviewees stated that these strategies are not specified in any official document. However, all providers and the majority of community participants were familiar with these two practices.

*“I don’t have the strategy on paper here. But we want the men to be involved. We have some already present during antenatal consultations and we would like them to be there for the delivery as well. We try to get the men to antenatal consultations by giving priority to couples.” Government officer, age group 40–50, key informant interviews.*

Most donors interviewed do not consider male involvement as a priority. The larger NGOs often focus on HIV and invest in mass campaigns with measurable outcomes. Experts explained that male involvement is not a topic that attracts donors, especially when there are so many other health problems on the national agenda such as HIV, malnutrition, malaria and intimate partner violence. The low interest at policy level is often explained by the fact that overall quality of care is low and the health care system already overwhelmed, so that there is limited appetite for initiatives that invite even more “clients” to health facilities.

*Personally I don’t think it’s a priority and there are many other problems in this country... I would rather do a mass HIV screening campaign for men at the border with South Africa than inviting them in the regular health system.” NGO officer, age group 50–60, key informant interviews.*

### Parental leave

Currently there are no clear policies regarding ANC attendance for men during working hours. Health centers are only open during working hours, which is perceived as a barrier for men. Some health centers do provide a medical certificate that the man can present to his employer, but others do not. Both community participants and providers were of the opinion that official parental leave for going with your wife with ANC would facilitate male involvement during pregnancy.

### Educational curriculum

Experts familiar with the training curriculum for Maternal and Child Health (MCH) nurses explained it is very focused on women and children, with no specific attention paid to the role of men or their specific health needs. Very often the MCH nurse is also in charge of general consultations and in many cases is the only person with a medical training in the health center. General health problems are addressed during their training but specific problems related to men’s health, such as inguinal hernia and prostate problems, receive very little attention. Moreover providers lack supportive supervision during their career once they complete their studies, limiting opportunities for continuous learning and skills improvement.

## Discussion

**Fig. 2:**
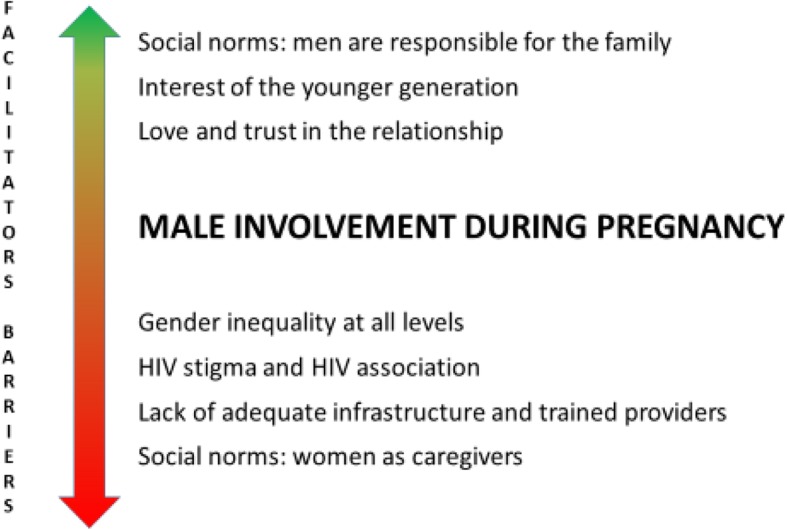
Key barriers and facilitators for male involvement during pregnancy in southern Mozambique

Our findings suggest the existence of strong social norms in southern Mozambique regarding the responsibility of men to take care of their wife and family within the community: while this is seen as very much part of men’s roles, there are also aspects that are seen as the women’s domain, particularly those relating to pregnancy and childbirth and including attending ANC (see Fig. 2). However younger men wanted to break with these traditions. Until now attending ANC with the wife during pregnancy is often not considered as “taking care of the family”. Studies in Kenya, Ghana and Uganda found similar results: traditionally men are supposed to take care of the family but very often pregnancy and childbirth is considered as the women’s domain [28–30]. In our study a generational evolution was noted regarding men’s interest in maternal health care issues. Younger men were generally more motivated to take an active role during pregnancy and even be present during antenatal consultations and delivery. However, barriers at the health facility and strong social norms within the community often deterred these young men from putting their intentions into practice. Interventions aimed at increasing male involvement should incorporate and build on these existing roles of men as caretakers of the family and could use this message to persuade men to attend ANC together with the wife.

Echoing the results of another study in central Mozambique [5], male attendance at ANC was associated with being weak and/or HIV positive in our study. This might be related to the fact that most male involvement programs in Mozambique and beyond have so far focused narrowly on men’s involvement in PMTCT and have generally not tackled male involvement in a broader sense [31, 32]. Male involvement is often limited to testing the male partner in addition to PMTCT [33]. This tendency has led to high stigmatization of men going with their wife to ANC. Policymakers, researchers and program managers working on HIV should be aware of this problem and focus more on promoting male involvement during pregnancy as an important aspect of taking care of their partner and unborn baby, rather than specifically as a strategy for strengthening HIV prevention and care.

At health facility level we observed several situations where the presence of the husband during ANC can have a negative impact on the empowerment of the woman. Due to persisting socio-cultural beliefs related to gender, when a man attends ANC, both the provider (because of their profession) and husband (because of gender inequality) will take a superior role, which places the woman in a submissive position. These power dynamics will affect the consultation in a negative way, in that sense that the woman will be less able to express her doubts, questions, needs or concerns. These dynamics were also described during the FGDs by providers and community members. To address this problem, providers could be trained to promote gender equity within the consultation. Research has shown that gender sensitive programs are more effective in changing health related behavior than narrowly focused interventions [14, 24, 34].

Our study demonstrates that some initiatives have been taken by the Mozambican government to involve men in ANC such as priority attendance for couples and distribution of partner invitation cards during ANC. However, they have apparently had minimal success in the study area, as most women continue to frequent ANC alone and most men do not consider it important to attend ANC with their pregnant partner. This way of program planning is also seen in other countries: male involvement interventions commonly adopt a reductionist and instrumentalist approach that is focused on altering men’s behaviours, without addressing underlying gender roles that drive these behaviours [14].

Instead of the priority rule for couples we would recommend governments invest more in high quality antenatal care consultations that are adapted to also receive couples. The current priority rule can be seen as a quick fix that is unfortunately also reinforcing gender inequality. In order to reduce waiting times for both women and couples at ANC, an appointment booking system (currently being piloted in Mozambique) could be considered [35, 36]. Another challenge when inviting male partners to ANC is offering privacy during the consultation, which is less of an issue with women only. However, investments in health infrastructure are hard to obtain in low resource settings where many other health priorities require attention.

Another important building block for high quality couple consultations will be the educational curriculum of providers working in primary care. Before inviting men to ANC, providers should be trained to deal with couples in an equal way and address the needs of both partners. Until now health centers have been very female oriented with a strong focus on maternal health, which could be shifted to more family-oriented care.

Sensitization of men is mainly done in the health facility or by passing health messages to men through their partners. Our findings also suggest that very little is done at community level and initiatives are concentrated at health facility level. We believe it would be more effective to target men directly in the places where they gather, for example in community meetings, churches, workplaces or other meeting points. By organizing health promotion talks and awareness raising events at community level for both men and women, maternal health could be understood as a shared responsibility and not that of women alone. Additionally, schools are important places to address underlying gender dynamics in the household and promote gender equality from an early age.

### Limitations

There are a number of methodological constraints that limit the interpretation and generalizability of this study. Participants were recruited by community leaders and participation was voluntary. This resulted in participants with a relatively high socio-economic status (illustrated for example by the fact that most of them had access to television) and an interest in the topic. Families with low access to health services were probably poorly represented. Also, specific cultural norms and socio-economic dynamics characterizing southern Mozambique may limit the extent to which the results from this study are generalizable to other regions. Another limitation is the different languages (Changana, Portuguese and English) used throughout the process from study participant to reader. Although all translations were double checked by bilingual members of the research team, translation is an interpretive act and some meaning may have been lost [37].

## Conclusion

Our study showed there is not a coherent strategy in Mozambique to promote male involvement during pregnancy. A significant structural problem is the lack of adequate health infrastructure and trained providers to deal with both the pregnant woman and her male partner during ANC. In the community strong positive social norms exist regarding the responsibility of men to take care of their wife and family. However, the association of men attending ANC with being HIV positive and strong gender roles keep them away from active involvement during the antenatal period. Social norms regarding parenting as a shared responsibility that starts before birth should be incorporated in male involvement strategies in maternal health instead of the current focus on HIV prevention and care. Gender-equitable relationships between men and women should be encouraged in all maternal health interventions and providers should be trained to deal with couples in ANC in an equal way.

## Supplementary information

**Additional file 1:** Checklist observations.

**Additional file 2:** Interview Guides.

## Data Availability

The datasets used and/or analyzed during the current study are available from the corresponding author upon reasonable request.

## Abbreviations

ANC: Antenatal care
ARV: Anti-retroviral medicine
CHW: Community health worker
FGD: Focus group discussion
HIV: Human immunodeficiency virus
KII: Key informant interviews
MCH: Maternal and child health
NGO: Non-governmental organisation
PMTCT: Prevention of Mother to Child Transmission of HIV
RQDA: R Qualitative Data Analysis
UEM: Universidade Eduardo Mondlane
WHO: The World Health Organization
