# Supplementary material for: Policymaker, health provider and community perspectives on male involvement during pregnancy in southern Mozambique: a qualitative study
Source: BMC Pregnancy Childbirth. 2019 Oct 28;19:384. doi: 10.1186/s12884-019-2530-1 (PMC6819364; doi:10.1186/s12884-019-2530-1)
Supplement: Supplementary file 1 — Additional file 1: Checklist observations. [file 12884_2019_2530_MOESM1_ESM.docx]

**Observation Number:**

**Consultation**

Place:

Number of ANCs:

Number of first ANC:

Number of follow up ANC

Convite given to first ANC :

Convite given to follow up visit :

Male provider :

Female provider:

**Quality of Care:**

Privacy:

Health care provider washed hands/used hand alcohol :

Health care provider measures bloodpressure & weight:

Palpation of abdomen + hartbeat with pinard + measuring:

Counting hartbeat :

HIV testing woman:

Other testing/procedures:

**Male involvement**

Man Present:

Man had a chair:

HIV testing man:

Other testing/procedures man:

Man was given health information:

Woman receives information (when man is present):

**Narrative Observation**
