# Supplementary material for: Policymaker, health provider and community perspectives on male involvement during pregnancy in southern Mozambique: a qualitative study
Source: BMC Pregnancy Childbirth. 2019 Oct 28;19:384. doi: 10.1186/s12884-019-2530-1 (PMC6819364; doi:10.1186/s12884-019-2530-1)
Supplement: Supplementary file 2 — Additional file 2: Interview Guides. [file 12884_2019_2530_MOESM2_ESM.docx]

**Individual interviews: IV Guide**

**Discussion**

Before we start the discussions please introduce yourself by telling me your nature of work and what you like most about your work.

How long have you been involved in maternal health care research/policy/education ?

**Interview**

**What does male involvement during pregnancy mean for you?**

*Would you please describe Male Involvement in antenatal care services in your own terms?*

*What is the relevance of male involvement in maternal health services?*

*Describe the current level and type of male involvement?*

*How many men roughly accompany their partners during ANC services?*

*What would be regarded as the desired level of male involvement in antenatal care according to you?*

*What kind of involvement do these men display?*

*How does culture and gender influence male involvement during pregnancy?*

**What are perceived benefits of greater male involvement during pregnancy?**

*What are some factors that would promote or facilitate MI?*

*What could be the benefits?*

**What are potential risks associated with greater male involvement during pregnancy?**

*What could be the risks or negative consequences?*

**What are perceived challenges to increasing male involvement during pregnancy?**

*What are some factors that make it difficult for MI?*

*Is MI a priority within maternal health?*

**What are the opportunities of greater male involvement during pregnancy?**

*What are some factors that would encourage a male partner to be more involved?*

*What are the resources that are needed in order to promote MI during ANC?*

*What are some of the strategies that may be used to promote MI during pregnancy?*

*Out of the strategies outlined, which one would be the best strategy to use and try out now?*

**FGDs Community : IV Guide**

**Roteiro de entrevista**

**O que significa o ‘Envolvimento do Homem’ para você?**

Poderia explicar o ‘Envolvimento do Homem’ durante a gravidez em suas próprias palavras?

Qual é a relevância do envolvimento do homem nos serviços de saúde materna?

Descreva o modo do envolvimento do homem e seu envolvimento atual?

Quantos homens acompanham mais ou menos suas parceiras durante as consultas pré-natais?

O que seria considerado como nível desejado do envolvimento do homem no atendimento pré-natal en sua opinião?

Que/Qual tipo de envolvimento seria?

Como a cultura influencia o envolvimento masculino durante a gravidez?

**Quais são os benefícios de um maior envolvimento do homem na saúde materna?**

Quais são fatores que promoveriam ou facilitariam o envolvimento do homem?

Quais poderiam ser os benefícios?

**Quais são os (outros) riscos potenciais (a mais) associados envolvimento do homem?**

Quais poderiam ser os riscos ou as consequências negativas?

**Quais são os desafios para o aumento do envolvimento do homem?**

Quais são alguns dos fatores que dificultam o envolvimento do homem?

O envolvimento do homem é uma prioridade na saúde materna?

**FGDs Providers : IV Guide**

**O que significa a gravidez para você?**

**Poderia descrevar o modo do envolviment do homem atual durante a gravidez?**

Decisão de freqüentar consultas pré-natais/local de nascimento/presença CPN/apoio financeiro e transporte/sabe aonde ir no caso de um problema ...

**Como a cultura influencia o envolvimento masculino durante a gravidez?**

Qual é a relevância do envolvimento do homem na gravidez?

Quantos homens acompanham mais ou menos suas parceiras durante as consultas pré-natais?

O que seria considerado como nível desejado do envolvimento do homem no atendimento pré-natal en sua opinião?

Que/Qual tipo de envolvimento seria?

**Acharia homens querem ser envolvidos?**

Quais são os benefícios de um maior envolvimento do homem na saúde materna?

**Quais são fatores que promoveriam ou facilitariam o envolvimento do homem?**

Quais poderiam ser os benefícios?

Quais são os (outros) riscos potenciais (a mais) associados envolvimento do homem?

Quais poderiam ser os riscos ou as consequências negativas?

**Quais são os desafios para o aumento do envolvimento do homem?**

Quais são alguns dos fatores que dificultam o envolvimento do homem?
